# Variable selection for estimating optimal treatment regimes with multiple treatments

**DOI:** 10.1038/s41598-026-55573-y

**Published:** 2026-06-14

**Authors:** Yuexin Fang, Yu Liu

**Affiliations:** Department of Mathematics, Shanghai Normal University, Shanghai, China

**Keywords:** Optimal treatment regime, High-dimensional, Variable selection, Doubly robust estimation, Multiclass classification, Computational biology and bioinformatics, Mathematics and computing, Medical research

## Abstract

We propose a penalized classification method for estimating optimal treatment regimes (OTRs) with multiple treatments when the number of covariates is large. Our approach reformulates the OTR estimation problem as a weighted multiclass classification problem and integrates variable selection with doubly robust estimation into a unified framework that simultaneously performs variable selection and regime estimation. By employing a data expansion technique and incorporating $L_1$-type penalization along with augmented inverse probability weighting (AIPW) estimators, the method effectively identifies the sparse subset of covariates that genuinely drive treatment effect heterogeneity. Extensive simulation studies demonstrate the superior performance of the proposed method in terms of accuracy and double robustness for estimating the optimal treatment regimes. The method’s practical utility is further illustrated through an application to a clinical trial for chronic depression.

## Introduction

Personalized medicine seeks to improve clinical outcomes by tailoring treatments to individual patient characteristics. A fundamental aspect of this approach involves estimating optimal treatment regimes (OTRs), which are decision rules that map patient information to treatment assignments to maximize the expected clinical outcome^1–5^. While substantial methodological progress has been made for binary treatment settings^6–8^, many clinical applications involve multiple treatment options. The extension from binary to multiple treatments introduces significant methodological challenges, as it requires simultaneous comparison of several treatment contrasts rather than a single contrast.

The problem becomes more complex when the number of potential predictors is large. Modern clinical studies often collect a large number of covariates, including genomic data, detailed clinical measurements, and demographic information. In such settings, variable selection becomes essential for several reasons. First, including all available covariates can lead to overfitting and poor generalization performance. Second, among the many measured variables, only a subset is likely to be relevant for treatment decision-making. Some covariates may be prognostic (predictive of outcome) but not prescriptive (modifying treatment effect), while others may be irrelevant noise. Identifying the sparse set of prescriptive variables is crucial for developing interpretable and clinically useful treatment rules.

Current methods for multi-treatment OTR estimation, such as Adaptive Contrast Weighted Learning^9^ and Tree-based Reinforcement Learning^10^, were developed for low-dimensional settings and lack built-in variable selection capabilities. When applied to data with a large number of covariates, these methods typically yield unstable estimates and suboptimal treatment rules. Multi-treatment OTR estimation has also been extended to survival outcomes^11,12^. For OTRs with a large number of covariates in binary treatment settings, doubly robust pseudo-weights have been incorporated into a penalized classification framework with variable selection^13^, and some other methods have also been developed^14–19^. For multi-treatment OTRs, variable selection with a large number of covariates remains less developed. Several recent works have explored this direction. Multicategory outcome-weighted margin-based learning with an $L_1$ penalty has been proposed for multi-treatment OTRs^20^. Angle-based learning has also been developed to simultaneously handle multiple treatments and perform feature selection^21^. However, both methods rely on inverse probability weighting (IPW) and therefore lack double robustness.

The OTR estimation problem was reformulated as a weighted classification problem for binary treatments, offering a convenient and interpretable framework which can be implemented with AIPW estimators to achieve double robustness^7^. However, its extension to multiple treatments with double robustness and built-in variable selection has received limited attention. This gap is particularly important because multi-treatment OTRs require estimating multiple contrast functions, each of which may depend on different subsets of covariates. To address this limitation, we extend the classification framework to multi-treatment settings with a large number of covariates, integrating doubly robust estimation and LASSO-based variable selection. Our key contributions are sparse doubly robust estimators for multi-treatment contrast functions and a unified optimization procedure that simultaneously performs variable selection and regime estimation. Through extensive simulation studies and a real-data application to a chronic depression trial, we demonstrate that the penalized extension significantly improves upon the unpenalized version in both variable selection accuracy and regime estimation performance. The proposed framework represents a substantial advance in personalized treatment.

The remainder of this paper is organized as follows. We first introduce the formal framework for high-dimensional multi-treatment OTRs. We then detail the proposed penalized classification methodology. Simulation studies and a real-data application are presented next, followed by concluding remarks.

## Notation and review of classification reformulation

We consider the problem of estimating an optimal treatment regime from data comprising *n* independent and identically distributed triplets $\{(X_i, A_i, Y_i)\}_{i=1}^n$. Here, $X_i \in \mathbb {R}^p$ represents a vector of patient covariates, which is often high-dimensional in modern applications. The treatment assignment $A_i$ takes values in a finite set $\mathcal {A} = \{0,1,\ldots ,K\}$, indicating $K+1$ treatment options. The observed outcome is denoted by $Y_i \in \mathbb {R}$, with the convention that larger values are more desirable.

Within the potential outcomes framework, we let $Y^*(a)$ signify the outcome that would be observed under treatment $a\in \mathcal {A}$. A treatment regime, *g*, is a deterministic function mapping the covariate space to the treatment space, i.e., $g:\mathbb {R}^p\rightarrow \mathcal {A}$. The performance of a regime *g* is measured by its value function, defined as the expected outcome under the regime: $V(g) = E[Y^*\{g(X)\}]$. The central goal is to find the optimal treatment regime $g^{\textrm{opt}}$ that maximizes this value: $g^{\textrm{opt}} = \arg \max _{g\in \mathcal {G}} V(g)$, where $\mathcal {G}$ denotes a predefined class of candidate regimes. In order for the optimal treatment regime to be identifiable from the observed data, we make the following standard assumptions:

(C1) SUTVA (Stable Unit Treatment Value Assumption): $Y=\sum _{k=0}^{K}I(A=k)Y^{*}(k)$;

(C2) No unmeasured confounder assumption (strong ignorability assumption): $A\bot {Y^{*}(a)}_{a\in \mathcal {A}}\mid X$;

(C3) Positivity: For each treatment $k \in \mathcal {A}$, the propensity score satisfies $0< \pi _k(X) < 1$ for almost every *X*.

Under these assumptions, the value function

$$V(g) = E_X\left[ E\{Y \mid X, A = g(X)\} \right] = E\left[ \mu \{g(X),X\} \right] ,$$

where $\mu (a, x) = E(Y \mid A=a, X=x)$ is the conditional mean function. Define the contrast functions $C_k(X) = \mu (k,X) - \mu (0,X)$ for $k=1,\ldots ,K$, with $C_0(X)\equiv 0$, then value function can be decomposed as:

$$V(g) = E\{ \mu (0, X) \} + E\left[ \sum _{k=1}^{K} I\{g(X) = k\} \cdot C_k(X) \right] .$$

Since the first term, $E\{\mu (0, X)\}$, is independent of the regime *g*, the problem of maximizing *V*(*g*) is equivalent to maximizing:

1 $$\begin{aligned} E\left[ \sum _{k=1}^{K} I\{g(X) = k\} \cdot C_k(X) \right] . \end{aligned}$$

As shown in Fang et al.^22^, the optimal treatment regime can be reformulated as a weighted classification problem via a data expansion technique^23^. Specifically, each original observation is expanded to $K+1$ copies $\{X_i, k, w_k(X_i)\}$ for $k = 0,\ldots ,K$, where $w_k(X) = C_k(X) - \min _j C_j(X)$. The optimal regime then satisfies:

2 $$\begin{aligned} g^{\text {opt}} = \arg \min _{g\in \mathcal {G}} E \left[ \sum _{k=0}^{K} w_k(X_i) \cdot I\{g(X_i) \ne k\}\right] . \end{aligned}$$

In practice, the contrast functions are unknown. Using AIPW estimators, we obtain $\widehat{C}_k(X_i)$ and construct $\widehat{w}_k(X_i)=\widehat{C}_k(X_i)-\min _j\widehat{C}_j(X_i)$. So we can estimate the optimal treatment regime by:

3 $$\begin{aligned} \widehat{g}^{\textrm{opt}} = \arg \min _{g\in \mathcal {G}} \frac{1}{n(K+1)} \sum _{i=1}^{n} \sum _{k=0}^{K} \widehat{w}_k(X_i) \cdot I\{g(X_i) \ne k\}. \end{aligned}$$

where

$$\widehat{C}_{k}(X_{i}) = \widehat{\mu }(k,X_{i}) - \widehat{\mu }(0,X_{i}) + \frac{I(A_{i}=k)}{\widehat{\pi }_{k}(X_{i})}\{Y_{i}-\widehat{\mu }(k,X_{i})\} - \frac{I(A_{i}=0)}{\widehat{\pi }_{0}(X_{i})}\{Y_{i}-\widehat{\mu }(0,X_{i})\}.$$

The doubly robust property of AIPW estimators ensures consistent estimation if either the propensity score model or the outcome regression model is correctly specified^24^. We assume a multinomial logistic model for the propensity scores and a linear model for the outcome regression, with sparsity enforced by LASSO penalties.

Thus, the problem is transformed into a weighted multiclass classification problem. Although this reformulation is easy to implement, its direct application to high-dimensional data faces two major challenges: (i) the 0–1 loss is nonconvex and nonsmooth; (ii) the class of possible regimes becomes too large when *p* is large, leading to overfitting. These limitations motivate the extension we propose in the next section.

## Penalized classification for high-dimensional OTRs

Building on the reformulation in (2), we recast the optimal regime estimation problem into a form that is directly amenable to smooth surrogate losses and standard regularization techniques. This enables us to propose a penalized classification method for high-dimensional data. We restrict consideration to linear rules of the form $g(X) = \arg \max _{k \in \mathcal {A}} {\eta }_k^\top H_X$, where $H_X$ is a basis expansion of the covariates *X* (e.g., including linear terms and possibly quadratic or interaction terms). Under this parameterization, the objective from (3) can be equivalently solved by fitting a weighted multinomial logistic regression model. For identifiability, we set treatment 0 as the reference category with ${\eta }_0 = {\textbf {0}}$. The model is specified through the log-odds:

$$\ln \left( \frac{P\{g(X)=k \mid X\}}{P\{g(X)=0 \mid X\}} \right) = {\eta }_k^\top H_X, \quad \text {for } k = 1, \ldots , K,$$

which gives the complete probability distribution:

$$P\{g(X)=k \mid X\} = {\left\{ \begin{array}{ll} \frac{1}{1 + \sum _{j=1}^{K} \exp ({\eta }_j^\top H_X)} & \text {for } k = 0,\\ \frac{\exp ({\eta }_k^\top H_X)}{1 + \sum _{j=1}^{K} \exp ({\eta }_j^\top H_X)} & \text {for } k = 1, \ldots , K. \end{array}\right. }$$

The connection to the original objective (3) is established through the weighted log-likelihood. For a parameter vector ${\eta }= ({\eta }_1^\top , \ldots , {\eta }_K^\top )^\top$, the weighted negative log-likelihood is:

4 $$\begin{aligned} \mathcal {L}_n({\eta }) = -\frac{1}{n(K+1)} \sum _{i=1}^{n} \sum _{k=1}^{K} \widehat{w}_k(X_i) \ln P\{g(X_i)=k \mid X_i\}. \end{aligned}$$

Minimizing $\mathcal {L}_n({\eta })$ is equivalent to maximizing the weighted likelihood and, by construction, to solving the empirical classification problem (2) under the linear decision rule family.

To handle high-dimensional covariates and achieve variable selection, we add an $L_1$ penalty term. The final objective function for our penalized estimator is:

5 $$\begin{aligned} \min _{{\eta }} \left\{ \mathcal {L}_n({\eta }) + \lambda \sum _{k=1}^{K} \Vert {\eta }_k\Vert _1 \right\} , \end{aligned}$$

where $\lambda \ge 0$ is a tuning parameter controlling the sparsity level, and $\Vert {\eta }_k\Vert _1 = \sum _{l=1}^p |{\eta }_{k,l}|$ is the $L_1$ norm.

The optimization problem (5) corresponds to fitting a weighted multinomial logistic regression with a Lasso penalty. A major practical advantage of this formulation is that it can be efficiently implemented using standard software packages (e.g., the glmnet package in R). The tuning parameter $\lambda$ can be selected via cross-validation. The final estimated optimal treatment regime is:

$$\widehat{g}^{\text {opt}}(X) = \arg \max _{k \in \mathcal {A}} \widehat{{\eta }}_{k}^{\top } H_X.$$

In this penalized classification approach, variable selection is naturally embedded through the LASSO penalty, which encourages sparsity, meaning that if a covariate (or its basis function) is unimportant for treatment decision-making, its corresponding coefficients across all treatment categories $(\eta _{1,l}, \dots , \eta _{K,l})$ will be shrunk to zero.

## Simulation studies

We conduct comprehensive simulation studies to evaluate the performance of the proposed method. The evaluation focuses on two primary aspects: (1) the accuracy of treatment decisions made by the estimated regime $\widehat{g}^{\text {opt}}$, measured via the error rate; (2) the efficacy of variable selection in identifying prescriptive variables. We consider scenarios with varying numbers of treatments (three treatments, $K=2$, and four treatments, $K=3$) and covariate dimensions ($p=10$ and $p=50$). For each scenario, we generate training datasets of size $n=500$ or $n=1000$ to estimate the optimal regime, and evaluate performance on an independent test set of size 1000, where the true optimal treatment is known. Each simulation configuration is repeated 500 times. We compare both the penalized and unpenalized versions to assess the benefit of incorporating the $L_{1}$-penalty. The *p*-dimensional covariate vector $X=(X_{1},\ldots ,X_{p})^{\top }$ has all components independently generated from *N*(0, 1). The first five variables $X_{1},X_{2},X_{3},X_{4},X_{5}$ are prescriptive variables that appear in the optimal treatment regime $g^{\text {opt}}(X)$; the remaining $p-5$ variables are noise variables. The error term $\epsilon \sim N(0,1)$. Propensity scores are estimated via a two-step procedure: first, LASSO-penalized multinomial regression for variable selection; second, unpenalized multinomial logistic regression on the selected variables. We consider three simulation scenarios:

**Scenario 1 (Linear regime,** $K=2$): Three treatments with $A\in \{0,1,2\}$. The propensity scores are $\pi _0(X)=1/\{1+\exp (0.5X_1+0.5X_4)+\exp (-0.5X_1+0.5X_5)\}$, $\pi _1(X)=\exp (0.5X_1+0.5X_4)\pi _0(X)$, $\pi _2(X)=1-\pi _0(X)-\pi _1(X)$. Consider four outcome models: (a) $Y=\exp [1.5+0.3X_4+\sum _{a=0}^{2}[I(A=a)\{2I(g^{\text {opt}}=a)-1\}]+\epsilon$; (b) $Y=\exp [1.5+0.3X_4-|1.5X_1-1|\cdot I\{A\ne g^{\text {opt}}(X)\}\{4I(A=0)+I(A=1)+2I(A=2)\}]+\epsilon$; (c) $Y=\exp (2+0.2X_3-|X_1+X_2|\cdot 3I\{A\ne g^{\text {opt}}(X)\})+\epsilon$; (d) $Y=\exp (2+0.2X_3-|X_1+X_2|\cdot \{A-g^{\text {opt}}(X)\}^2)+\epsilon$. The optimal regime is linear: $g^{\text {opt}}(X)=\arg \max _{k\in \{0,1,2\}}{\eta }_k^{\top }X$, with ${\eta }_0=\textbf{0}_p$, ${\eta }_1=(1,-1,\textbf{0}_{p-2})^{\top }$, ${\eta }_2=(1,-1,1,\textbf{0}_{p-3})^{\top }$, where $\textbf{0}_q$ denotes the *q*-dimensional zero vector.

**Scenario 2 (Nonlinear regime,** $K=2$): Treatment and outcome generation are identical to those in Scenario 1. The optimal regime is nonlinear: $g^{\text {opt}}(X)=\arg \max _{k\in \{0,1,2\}}{\eta }_k^{\top }H_X$, with ${\eta }_0^{\top }H_X=0$, ${\eta }_1^{\top }H_X=X_1-X_2^2$, ${\eta }_2^{\top }H_X=X_1-X_2^2+X_3^2$, and $H_X=(X_1,X_2^2,X_3^2)^{\top }$.

**Scenario 3 (Linear regime,** $K=3$): Four treatments with $A\in \{0,1,2,3\}$. The propensity scores are $\pi _0=1$, $\pi _1=\exp (0.5-0.5X_1)$, $\pi _2=\exp (0.5X_1+0.2)$, $\pi _3=\exp (0.5X_5+0.1)$, $\pi _s=\sum _{m=0}^{3}\pi _m$, and $\pi _k(X)=\pi _k/\pi _s$ for $k=0,\ldots ,3$. The outcome models are (a) $Y=\exp [1.5+0.3X_4+\sum _{a=0}^{3}[I(A=a)\{2I(g^{\text {opt}}=a)-1\}]+\epsilon$; (b) $Y=\exp [1.5+0.3X_4-|1.5X_1-1|\cdot I\{A\ne g^{\text {opt}}(X)\}\{4I(A=0)+I(A=1)+2I(A=2)+I(A=3)\}]+\epsilon$; (c) and (d) are identical to Scenario 1. The optimal regime is linear: $g^{\text {opt}}(X)=\arg \max _{k\in \{0,1,2,3\}}{\eta }_k^{\top }X$, with ${\eta }_0=\textbf{0}_p$, ${\eta }_1=(1,-1,\textbf{0}_{p-2})^{\top }$, ${\eta }_2=(1,-0.5,0.5,\textbf{0}_{p-3})^{\top }$, ${\eta }_3=(1,0,-0.5,0,0.5,\textbf{0}_{p-5})^{\top }$.

**Scenario 4 (Misspecified propensity score,** $K=2$): Three treatments with $A\in \{0,1,2\}$. The propensity scores are $\pi _0=1$, $\pi _1=\exp (0.5X_4+0.5X_1^2)$, $\pi _2=\exp (0.5X_5^2-0.5X_1)$, $\pi _s=\sum _{m=0}^{2}\pi _m$, and $\pi _k(X)=\pi _k/\pi _s$ for $k=0,\ldots ,2$. The propensity score model used for estimation includes only linear terms (without $X_1^2$ and $X_5^2$), thus misspecified. $Y = 1 + X_4+ X_5 + 2I(A=1)(X_1-X_2) + 2I(A=2)(X_1-X_2+X_3) + \epsilon$, where $\epsilon \sim N(0,1)$. So the optimal treatment regime is the same as in Scenario 1.

Performance is evaluated using the error rate (ER) of treatment decisions: $\text {ER}=n^{-1}\sum _{i=1}^{n}I\{\widehat{g}^{\text {opt}}(X_i)\ne g^{\text {opt}}(X_i)\}$, and the value ratio (VR): $\text {VR}=V(\widehat{g}^{\text {opt}})/V(g^{\text {opt}})$, where $V(g)=E[Y^*\{g(X)\}]$ is computed using Monte Carlo integration. For penalized methods, we also report variable selection metrics: Size (the empirical mean of total number of selected variables), TP (the empirical mean of correctly selected prescriptive variables), and FP (the empirical mean of incorrectly selected noise variables). The true prescriptive set $S^*$ (the set of covariates that appear in the optimal treatment regime $g^{\text {opt}}(X)$) is defined at the feature level and differs by scenario. In Scenario 1, the optimal regime is linear in *X*, so $S^* = \{X_1, X_2, X_3\}$. In Scenario 2, the regime is nonlinear in the basis $H_X = (X_1, X_2^2, X_3^2)^\top$. The truly active features are $S^* = \{X_1, X_2^2, X_3^2\}$. For variable selection in Scenario 2, the candidate set includes all linear terms $X_1,\ldots ,X_p$ and all quadratic terms $X_1^2,\ldots ,X_p^2$, totaling 2*p* candidate variables. In Scenario 3, the optimal linear regime additionally involves $X_5$, giving $S^* = \{X_1, X_2, X_3, X_5\}$. The TP metric counts the number of features in the respective $S^*$ are assigned non-zero coefficients by the penalized estimator. An ideal selector would yield TP close to $|S^*|$ (i.e., 3, 3, and 4 for Scenarios 1, 2, and 3, respectively). For both penalized and unpenalized methods, we included quadratic terms of covariates when fitting models for Scenario 2 (nonlinear regime), while only linear terms were considered for Scenarios 1 and 3 (linear regimes). The tuning parameter $\lambda$ for the penalized method was selected through 10-fold cross-validation using the default settings in the glmnet package in R.

**Fig. 1:**
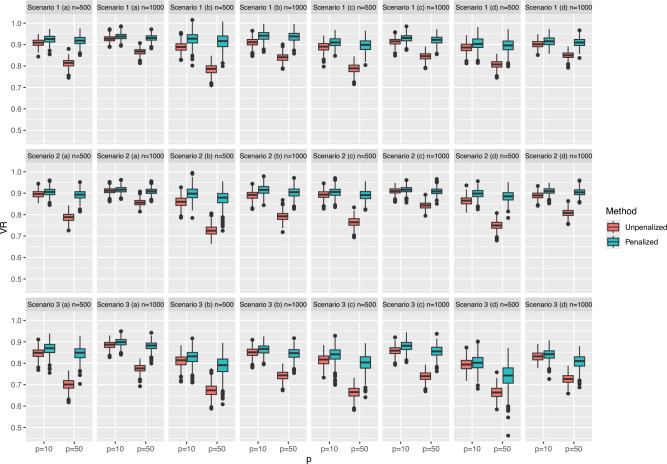
The error rate (ER) of the estimated treatment decisions under different methods and settings.

**Fig. 2:**
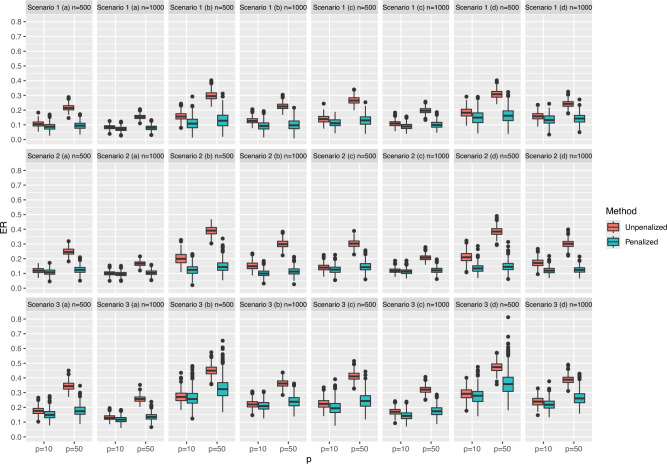
The value ratio (VR) under different methods and settings.

The simulation results presented in Tables 1–4 comprehensively demonstrate the performance of the proposed penalized method across various data-generating mechanisms, dimensions, and sample sizes. The analysis reveals several consistent patterns that highlight the advantages of incorporating $L_1$-penalization within the multi-treatment classification framework. In settings with a large number of covariates ($p = 50$), the penalized method substantially outperforms its unpenalized versions in both regime estimation accuracy and variable selection precision. For instance, under Scenario 1 (linear optimal regime, Table 1), the penalized method achieves lower ER and higher VR compared to the unpenalized version, while correctly selecting all true prescriptive variables (TP = 3). Similar improvements are observed under Scenario 2 (nonlinear optimal regime, Table 2) and Scenario 3 (four treatments, Table 3), where the penalized method consistently outperforms the unpenalized approach in both ER and VR. Across all scenarios, increasing the sample size from $n = 500$ to $n = 1000$ improves performance for both methods. The corresponding findings are visually apparent in Figs. 1 and 2. In Scenario 4, where the propensity score model is misspecified while the outcome model is correctly specified, the penalized method also achieves high VR and low ER (Table 4), illustrating the double robustness property.

These results illustrate the necessity of integrating variable selection with regime estimation in high-dimensional multi-treatment settings. The proposed penalized framework effectively addresses the high-dimensional challenge, maintains the double robust property through augmented inverse probability weighting, and yields interpretable treatment rules based on a sparse set of prescriptive variables. The consistent performance across different numbers of treatments, linear and nonlinear regimes, and different outcome models confirms the robustness and practical utility of the proposed methodology.

**Table 1:** Simulation results for Scenario 1 with three treatment options and the optimal regime is linear in covariates. (a), (b), (c), and (d) are the models for generating outcome variables, respectively. ER: the empirical mean and standard deviation (SD) of the error rate of decisions made by the estimated treatment regimes. VR (value ratio): the empirical mean and standard deviation (SD) of the ratio of the value of the estimated regime relative to that of the true optimal regime, where value is the expectation of potential outcomes; Size: the empirical mean of total number of selected variables; TP: the empirical mean of correctly selected prescriptive variables; FP: the empirical mean of incorrectly selected noise variables.

	Model	n	Unpenalized		Penalized				
			ER	VR	Size	TP	FP	ER	VR
$p=10$	(a)	500	0.106(0.019)	0.908(0.017)	5.60	3	2.60	0.087(0.022)	0.925(0.020)
		1000	0.084(0.014)	0.927(0.014)	5.23	3	2.23	0.072(0.016)	0.938(0.015)
	(b)	500	0.158(0.029)	0.889(0.023)	4.26	3	1.26	0.111(0.043)	0.926(0.030)
		1000	0.128(0.022)	0.911(0.019)	4.32	3	1.32	0.094(0.032)	0.940(0.023)
	(c)	500	0.139(0.026)	0.890(0.022)	5.60	3	2.60	0.114(0.030)	0.910(0.024)
		1000	0.109(0.019)	0.914(0.016)	5.48	3	2.48	0.090(0.021)	0.930(0.018)
	(d)	500	0.184(0.033)	0.885(0.022)	4.44	3	1.44	0.149(0.044)	0.905(0.028)
		1000	0.159(0.025)	0.902(0.017)	4.03	3	1.03	0.135(0.034)	0.916(0.021)
$p=50$	(a)	500	0.216(0.021)	0.813(0.019)	5.84	3	2.84	0.095(0.025)	0.917(0.022)
		1000	0.154(0.016)	0.866(0.015)	6.10	3	3.10	0.080(0.017)	0.931(0.015)
	(b)	500	0.297(0.029)	0.785(0.024)	4.73	3	1.73	0.131(0.052)	0.914(0.035)
		1000	0.225(0.022)	0.839(0.019)	4.19	3	1.19	0.100(0.036)	0.937(0.023)
	(c)	500	0.266(0.025)	0.788(0.022)	6.36	3	3.36	0.130(0.036)	0.898(0.028)
		1000	0.197(0.020)	0.845(0.018)	6.09	3	3.09	0.101(0.024)	0.921(0.019)
	(d)	500	0.309(0.029)	0.807(0.019)	4.26	3	1.26	0.163(0.052)	0.895(0.032)
		1000	0.242(0.024)	0.850(0.016)	4.13	3	1.13	0.142(0.033)	0.910(0.021)

**Table 2:** Simulation results for Scenario 2 with three treatment options and the optimal regime is nonlinear in covariates. The candidate set includes 2*p* variables (linear and quadratic terms). (a), (b), (c), and (d) are the models for generating outcome variables, respectively. ER: the empirical mean and standard deviation (SD) of the error rate of decisions made by the estimated treatment regimes. VR (value ratio): the empirical mean and standard deviation (SD) of the ratio of the value of the estimated regime relative to that of the true optimal regime, where value is the expectation of potential outcomes; Size: the empirical mean of total number of selected variables; TP: the empirical mean of correctly selected prescriptive variables; FP: the empirical mean of incorrectly selected noise variables.

	Model	n	Unpenalized		Penalized				
			ER	VR	Size	TP	FP	ER	VR
$p=10$	(a)	500	0.119(0.019)	0.896(0.017)	7.94	3	4.94	0.109(0.021)	0.905(0.019)
		1000	0.102(0.015)	0.910(0.014)	8.97	3	5.97	0.097(0.016)	0.916(0.015)
	(b)	500	0.202(0.037)	0.859(0.025)	7.59	3	4.59	0.125(0.034)	0.899(0.030)
		1000	0.152(0.026)	0.890(0.020)	9.20	3	6.20	0.102(0.023)	0.915(0.023)
	(c)	500	0.141(0.023)	0.893(0.019)	8.17	3	5.17	0.127(0.027)	0.905(0.021)
		1000	0.120(0.018)	0.910(0.015)	8.97	3	5.97	0.113(0.019)	0.916(0.016)
	(d)	500	0.213(0.036)	0.864(0.020)	6.14	3	3.14	0.136(0.030)	0.898(0.021)
		1000	0.172(0.028)	0.889(0.016)	6.87	3	3.87	0.121(0.023)	0.910(0.016)
$p=50$	(a)	500	0.248(0.023)	0.787(0.021)	6.73	3	3.73	0.125(0.025)	0.892(0.023)
		1000	0.167(0.017)	0.856(0.016)	6.98	3	3.98	0.105(0.017)	0.909(0.015)
	(b)	500	0.391(0.030)	0.725(0.024)	8.28	3	5.28	0.149(0.039)	0.877(0.035)
		1000	0.301(0.027)	0.792(0.021)	9.82	3	6.82	0.114(0.026)	0.903(0.024)
	(c)	500	0.303(0.027)	0.765(0.023)	7.21	3	4.21	0.146(0.032)	0.891(0.025)
		1000	0.207(0.020)	0.843(0.017)	8.04	3	5.04	0.122(0.021)	0.908(0.016)
	(d)	500	0.386(0.030)	0.749(0.021)	6.34	3	3.34	0.148(0.034)	0.886(0.025)
		1000	0.301(0.026)	0.808(0.017)	7.51	3	4.51	0.125(0.022)	0.904(0.017)

**Table 3:** Simulation results for Scenario 3 with four treatment options and the optimal regime is linear in covariates. (a), (b), (c), and (d) are the models for generating outcome variables, respectively. ER: the empirical mean and standard deviation (SD) of the error rate of decisions made by the estimated treatment regimes. VR (value ratio): the empirical mean and standard deviation (SD) of the ratio of the value of the estimated regime relative to that of the true optimal regime, where value is the expectation of potential outcomes; Size: the empirical mean of total number of selected variables; TP: the empirical mean of correctly selected prescriptive variables; FP: the empirical mean of incorrectly selected noise variables.

	Model	n	Unpenalized		Penalized				
			ER	VR	Size	TP	FP	ER	VR
$p=10$	(a)	500	0.177(0.026)	0.846(0.023)	6.58	4	2.58	0.153(0.032)	0.867(0.028)
		1000	0.131(0.019)	0.886(0.017)	6.99	4	2.99	0.117(0.022)	0.898(0.020)
	(b)	500	0.273(0.037)	0.811(0.028)	6.59	3.90	2.69	0.265(0.052)	0.828(0.034)
		1000	0.222(0.028)	0.851(0.022)	6.79	3.99	2.80	0.211(0.034)	0.865(0.024)
	(c)	500	0.224(0.033)	0.816(0.027)	6.91	3.99	2.92	0.197(0.044)	0.839(0.035)
		1000	0.171(0.024)	0.859(0.021)	6.84	4	2.84	0.145(0.028)	0.880(0.023)
	(d)	500	0.292(0.038)	0.794(0.028)	7.49	3.99	3.50	0.277(0.052)	0.802(0.036)
		1000	0.240(0.030)	0.832(0.022)	7.47	4	3.47	0.222(0.036)	0.841(0.026)
$p=50$	(a)	500	0.345(0.029)	0.701(0.026)	7.54	3.98	3.56	0.178(0.037)	0.845(0.032)
		1000	0.257(0.021)	0.777(0.019)	7.32	4	3.32	0.136(0.023)	0.882(0.022)
	(b)	500	0.449(0.034)	0.675(0.029)	7.00	3.62	3.38	0.330(0.072)	0.786(0.046)
		1000	0.363(0.026)	0.742(0.022)	7.52	3.97	3.55	0.241(0.042)	0.846(0.026)
	(c)	500	0.412(0.031)	0.665(0.027)	7.66	3.85	3.81	0.246(0.051)	0.802(0.041)
		1000	0.320(0.025)	0.740(0.022)	7.74	3.99	3.75	0.175(0.034)	0.855(0.027)
	(d)	500	0.473(0.034)	0.664(0.027)	8.49	3.86	4.63	0.367(0.084)	0.738(0.057)
		1000	0.388(0.029)	0.727(0.023)	9.05	3.99	5.06	0.265(0.046)	0.808(0.032)

**Table 4:** Simulation results for Scenario 4 (misspecified propensity score) with three treatment options. ER: the empirical mean and standard deviation (SD) of the error rate; VR (value ratio): the empirical mean and standard deviation (SD) of the ratio of the value of the estimated regime relative to that of the true optimal regime; Size: the empirical mean of total number of selected variables; TP: the empirical mean of correctly selected prescriptive variables; FP: the empirical mean of incorrectly selected noise variables.

	n	Unpenalized		Penalized				
		ER	VR	Size	TP	FP	ER	VR
$p=10$	500	0.109(0.020)	0.984(0.019)	3.38	3	0.38	0.074(0.029)	0.994(0.018)
	1000	0.093(0.016)	0.989(0.017)	3.37	3	0.37	0.066(0.025)	0.996(0.018)
$p=50$	500	0.197(0.019)	0.947(0.019)	3.25	3	0.25	0.077(0.031)	0.992(0.018)
	1000	0.147(0.017)	0.971(0.018)	3.19	3	0.19	0.065(0.027)	0.994(0.018)

## Application to real data

We apply the proposed method to data from a randomized clinical trial for chronic depression. The study compared three treatments for chronic depression: (1) cognitive behavioral therapy (CBT, treatment 0), (2) nefazodone (NEF, treatment 1), and (3) their combination (NEF + CBT, treatment 2). A total of 577 patients completed the 12-week follow-up, with the primary outcome being the score on the 24-item Hamilton Rating Scale for Depression (HRSD) at week 12. Lower HRSD scores indicate lower depression severity and thus better outcomes. To align with our methodological framework where larger outcome values are preferable, we define the transformed outcome as $Y = -\text {HRSD}$. This transformation ensures that maximizing the expected outcome corresponds to minimizing depression severity.

Based on previous analyses, we consider 50 baseline covariates that may influence the outcome, including demographic characteristics, clinical history, and biomarker profiles. All continuous covariates were standardized to zero mean and unit variance. Given the randomized design of the trial, the propensity scores $\pi _{k}(X)$ can be estimated directly by $\sum _{i=1}^{n}I(A_i=k)/n, k=0,1,2$ for all *X*. For the conditional mean models $\mu (a, X) = E(Y \mid A = a, X)$, we employ linear regression with LASSO penalty for variable selection. Table 5 compares treatment assignments and estimated values across methods. The unpenalized method assigns 71 patients to CBT, 117 to NEF, and 389 to combination therapy. The penalized method assigns more patients to combination therapy (461 patients), suggesting this treatment may be optimal for a larger subgroup when accounting for variable selection. The estimated values, computed via inverse probability weighting (with standard deviations in parentheses from 500 bootstraps), show that the penalized method achieves the highest value of $-8.17 (0.860)$, compared to $-8.83 (0.826)$ for unpenalized method, $-9.90 (0.552)$ for assigning all patients to combination therapy, $-14.79 (0.741)$ for assigning all patients to CBT, and $-14.94 (0.704)$ for assigning all patients to NEF. Although no formal inference is drawn due to sample size limitations, the point estimates suggest potential improvement from personalized treatment assignment based on selected covariates.

**Table 5:** Treatment assignments and estimated values (with standard deviations in parentheses, obtained via 500 bootstraps) in the depression study.

Method	Patients assigned			Estimated value (SD)
	CBT	NEF	NEF + CBT	
All to CBT	577	0	0	$-14.79$(0.741)
All to NEF	0	577	0	$-14.94$(0.704)
All to NEF + CBT	0	0	577	$-9.90$(0.552)
Unpenalized	71	117	389	$-8.83$(0.826)
Penalized	41	75	461	$-8.17$(0.860)

This application demonstrates the practical utility of penalized method in a realistic clinical setting. The method successfully identifies a sparse set of prescriptive variables while estimating an interpretable treatment regime. The fact that different covariates appear in different decision functions supports our theoretical motivation: multi-treatment OTRs require estimating multiple contrast functions, each potentially depending on different covariate subsets. The increased assignment to combination therapy under penalized method, coupled with the higher estimated value, suggests that variable selection helps identify patients who particularly benefit from the combination treatment.

## Discussion

In this paper, we have developed a penalized extension of a classification-based approach for estimating optimal treatment regimes (OTRs) with multiple treatments and high-dimensional covariates. This unified method integrates doubly robust estimation of treatment contrast functions with $L_1$-penalized multinomial logistic regression, enabling simultaneous variable selection and regime estimation.

A key methodological innovation is the incorporation of LASSO-type regularization into the weighted multiclass classification framework, enabling the identification of a sparse set of prescriptive variables that genuinely drive treatment effect heterogeneity, while effectively excluding noise variables. Our extensive simulation studies demonstrate that the proposed method maintains the double robustness property while achieving superior performance in both regime estimation accuracy and variable selection precision. Several features of the proposed framework contribute to its practical utility. First, the use of augmented inverse probability weighted estimation (AIPWE) for contrast functions ensures robustness against model misspecification. Second, the data expansion technique transforms the complex treatment selection problem into a standard weighted classification task, which facilitates implementation using established software packages like glmnet.

Despite these advances, several important directions remain for future research. First, while our current implementation focuses on linear decision rules, extending the framework to accommodate nonlinear and interaction effects through basis expansions or kernel methods would enhance its flexibility. Second, the extension to dynamic treatment regimes with multiple decision points represents a natural and important direction for chronic disease management. Third, investigating the performance of the proposed framework with ultra-high dimensional data (where $p \gg n$) and developing theoretical guarantees under such conditions would further broaden its applicability. Fourth, several recent works have extended multi-treatment OTR estimation to survival outcomes. Survival contrast-learning using SVM^11^ and Cox-based approaches with AIPW estimation^12^ have been developed. Our approach focuses on continuous outcomes but offers two distinct advantages: (i) it provides double robustness through AIPW weighting within a unified classification framework, and (ii) it performs built-in variable selection via LASSO, which is particularly valuable for identifying prescriptive variables. Extending our framework to handle survival outcomes and competing risks while preserving these advantages is an important direction for future research.

In conclusion, the proposed framework provides a robust and computationally efficient approach for estimating optimal treatment regimes in high-dimensional, multi-treatment settings. By integrating doubly robust estimation with regularized classification, it offers a principled solution for personalized treatment planning in data-rich clinical environments.

## Data Availability

The data presented in this study are available on reasonable request from the corresponding author.

## Appendix A: Additional Simulation Results

This section provides additional simulation results comparing our penalized method with Tree-based Reinforcement Learning (T-RL)^10^. The comparison was conducted under the following settings: Scenario 1 (linear optimal regime), Scenario 2 (nonlinear optimal regime), Scenario 3 (four treatments), and Scenario 4 (misspecified propensity score), with $p=50$ and $n=500$ (Table 6).

**Table 6:** Results of T-RL method under representative settings with $p=50$, $n=500$.

Scenario	Model	ER	VR
Scenario 1	(a)	0.182(0.025)	0.843(0.022)
	(b)	0.225(0.041)	0.825(0.030)
	(c)	0.299(0.084)	0.763(0.060)
	(d)	0.324(0.080)	0.785(0.043)
Scenario 2	(a)	0.202(0.036)	0.825(0.031)
	(b)	0.276(0.062)	0.800(0.038)
	(c)	0.229(0.045)	0.825(0.034)
	(d)	0.243(0.052)	0.811(0.037)
Scenario 3	(a)	0.299(0.045)	0.741(0.039)
	(b)	0.392(0.063)	0.736(0.044)
	(c)	0.384(0.087)	0.691(0.069)
	(d)	0.456(0.089)	0.678(0.062)
Scenario 4	-	0.307(0.049)	0.760(0.070)
